# Development and evaluation of a clinical model for lung cancer patients using stereotactic body radiotherapy (SBRT) within a knowledge‐based algorithm for treatment planning

**DOI:** 10.1120/jacmp.v17i6.6429

**Published:** 2016-11-08

**Authors:** Karen Chin Snyder, Jinkoo Kim, Anne Reding, Corey Fraser, James Gordon, Munther Ajlouni, Benjamin Movsas, Indrin J. Chetty

**Affiliations:** ^1^ Department of Radiation Oncology Henry Ford Health System Detroit MI USA

**Keywords:** treatment planning, lung, SBRT, knowledge‐based model

## Abstract

The purpose of this study was to describe the development of a clinical model for lung cancer patients treated with stereotactic body radiotherapy (SBRT) within a knowledge‐based algorithm for treatment planning, and to evaluate the model performance and applicability to different planning techniques, tumor locations, and beam arrangements. 105 SBRT plans for lung cancer patients previously treated at our institution were included in the development of the knowledge‐based model (KBM). The KBM was trained with a combination of IMRT, VMAT, and 3D CRT techniques. Model performance was validated with 25 cases, for both IMRT and VMAT. The full KBM encompassed lesions located centrally vs. peripherally (43:62), upper vs. lower (62:43), and anterior vs. posterior (60:45). Four separate sub‐KBMs were created based on tumor location. Results were compared with the full KBM to evaluate its robustness. Beam templates were used in conjunction with the optimizer to evaluate the model's ability to handle suboptimal beam placements. Dose differences to organs‐at‐risk (OAR) were evaluated between the plans generated by each KBM. Knowledge‐based plans (KBPs) were comparable to clinical plans with respect to target conformity and OAR doses. The KBPs resulted in a lower maximum spinal cord dose by 1.0±1.6Gy compared to clinical plans, p=0.007. Sub‐KBMs split according to tumor location did not produce significantly better DVH estimates compared to the full KBM. For central lesions, compared to the full KBM, the peripheral sub‐KBM resulted in lower dose to 0.035 cc and 5 cc of the esophagus, both by 0.4Gy±0.8Gy, p=0.025. For all lesions, compared to the full KBM, the posterior sub‐KBM resulted in higher dose to 0.035 cc, 0.35 cc, and 1.2 cc of the spinal cord by 0.2±0.4Gy, p=0.01. Plans using template beam arrangements met target and OAR criteria, with an increase noted in maximum heart dose (1.2±2.2Gy, p=0.01) and GI (0.2±0.4, p=0.01) for the nine‐field plans relative to KBPs planned with custom beam angles. A knowledge‐based model for lung SBRT consisting of multiple treatment modalities and lesion locations produced comparable plan quality to clinical plans. With proper training and validation, a robust KBM can be created that encompasses both IMRT and VMAT techniques, as well as different lesion locations.

PACS number(s): 87.55de, 87.55kh, 87.53Ly

## I. INTRODUCTION

Stereotactic body radiotherapy (SBRT) has been shown to be an effective treatment method of medically inoperable, early stage, non‐small cell lung cancer (NSCLC).^1^, ^2^, ^3^ Several groups have shown the efficacy of lung SBRT, using a combination of different immobilization and planning techniques, including static field techniques such as 3D conformal and intensity‐modulated radiotherapy (IMRT), as well as rotational techniques such as dynamic conformal arc and volumetric‐arc radiotherapy (VMAT). Each technique has its own advantages and disadvantages;^4^, ^5^, ^6^ however, all of these planning techniques require skill and experience in order to create an optimal plan that achieves tumor conformity and geometric organ sparing.

IMRT is often used to treat lung SBRT, providing conformal tumor coverage while minimizing dose to surrounding organs at risk (OAR). Although IMRT optimization can be more forgiving to suboptimal beam placement and less user‐dependent than forward planning, variability in IMRT planning still occurs and may be dependent on a planner's skill.^(7)^ Several groups have been working to improve IMRT plan quality by correlating patient geometric factors with dose distributions from previous plans.^8^, ^9^, ^10^, ^11^ Models can be generated from the mathematical correlation of the geometry and dose data from each patient plan and be used to predict dose‐volume histograms for a new patient geometry.^12^, ^13^

A knowledge‐based dose‐volume histogram (DVH) algorithm that estimates the DVH, then automatically generates objectives given patient and beam geometry, has been introduced commercially (RapidPlan v13.5, Varian Medical Systems, Palo Alto, CA). The knowledge‐based model (KBM) is built from existing clinical plans that have ideal tumor coverage and OAR doses. The KBM implemented within RapidPlan utilizes a principle component analysis (PCA)‐based approach, in which the first and second order principle components are determined to account for variation in DVH parameters.^(11)^ Previous groups have created and evaluated knowledge‐based models for head and neck^(14)^ and advanced hepatocellular cancer.^(15)^ Their results showed that knowledge‐based plans were comparable in plan quality to clinical plans and improved pass rates of clinical objectives. In this study a KBM, within the RapidPlan software, was built based on treatment plans for lung cancer patients treated with SBRT. Applicability of the KBM to variable treatment techniques, beam arrangements, and tumor location was evaluated.

## II. MATERIALS AND METHODS

### A. Patient plan characteristics and selection

All plans in this study were contoured using a 4DITV method to account for respiratory motion. All patients received a four‐dimensional CT (4D CT) in which the internal tumor volume (ITV) was generated from the maximum intensity projection (MIP).^(16)^ Contouring and planning was performed on the average CT postprocessed from the 4D CT. Planning tumor volumes (PTV) were expanded from the ITV using a uniform 3 mm margin for central lesions, and a 6 mm uniform margin for peripheral lesions. The PTV margins were manually modified by the physician in order to respect anatomical boundaries and avoid overlap between the PTV and critical OARs, such as the esophagus, heart, and brachial plexus. Overlap between the PTV and rib was ignored and the dose limit to the rib was often exceeded to achieve PTV dose coverage. Plans were prescribed to either 48 Gy or 36 Gy, 12 Gy per fraction. OARs were contoured and planned using dose constraints similar to RTOG 0915^(17)^ with slight modifications (Table 1). Plans were optimized in the Eclipse treatment planning system (Varian Medical Systems), using the anisotropic analytical algorithm (AAA) for final dose calculation. Typical beam setup for both IMRT and VMAT techniques used 6 MV, coplanar fields avoiding beams entering through the contralateral lung. IMRT plans consisted of 7–9 beams with 20°–35° between each field. VMAT plans consisted of two continuous partial arcs, approximately 120°–160° in length. The start and stop gantry positions varied with patient geometry and lesion location. VMAT collimator angles were mirrored between the two partial arcs, where default collimator angles of 30° and 330° were utilized.

**Table 1:** OAR dose constraints used for lung SBRT model for 12 Gy/fx plan in either 3 or 4 fractions

*Organ*	*Volume*	*Volume Max*.	*Max. Point Dose (Gy)* ^a^
Spinal Cord	<0.35 cc	20.8 Gy	26 Gy
		(5.2 Gy/fx)	(6.5 Gy/fx)
	<1.2 cc	13.6 Gy	
		(3.4 Gy/fx)	
Esophagus	<5 cc	18.8 Gy	30 Gy
		(4.7 Gy/fx)	(7.5 Gy/fx)
Heart	<15 cc	28 Gy	34 Gy
		(7 Gy/fx)	(8.5 Gy/fx)
Total Lung ‐ PTV	<10%	20 Gy	NA
Ribs			<105% Rx
Brachial Plexus	<3 cc	23.6 Gy	27.2 Gy
		(5.9 Gy/fx)	(6.8 Gy/fx)
Stomach	<10 cc	17.6 Gy	27.2 Gy
		(4.4 Gy/fx)	(6.8 Gy/fx)
Trachea	<4 cc	15.6 Gy	34.8 Gy
		(3.9 Gy/fx)	(8.7 Gy/fx)
Skin	<10 cc	33.2 Gy	36 Gy
		(8.3 Gy/fx)	(9 Gy/fx)

a Maximum point dose defined as 0.035 cc.

One hundred and five early stage lung SBRT plans previously treated at our institution were used in the model. All patients included in the model were included in an IRB‐approved protocol at Henry Ford Hospital. From the database of lung patients treated with SBRT at our institution, plans were chosen that were considered well planned and met all OAR constraints. The 105 plans consisted of 97 IMRT, 6 VMAT, and 2 3D CRT plans. A large percentage of the lung SBRT plans treated at our institution were IMRT, and is reflected by the large percentage of IMRT cases included the model. Two 3D CRT cases were small peripherally located lesions, which were included to increase the range of tumor volumes accounted for in the model. Since the lesions were peripherally located, they were fairly isolated from the OARs and had minor impact on the dose estimation in the high‐dose regions of the OAR's DVH.

Previous studies comparing IMRT and VMAT treatment planning for lung SBRT have found comparable PTV dose coverage and OAR dose sparing between the two treatment techniques.^5^, ^6^ Since the KBM correlates the geometrical relationship of the PTV and OAR to the DVH, due to the similarities in plan quality, IMRT and VMAT plans were included in a single model. Furthermore, an intertechnique, interinstitutional study for head and neck IMRT OAR modeling found that, if the same clinical criteria is used, a model can be interchanged between different treatment modalities such as IMRT and helical tomotherapy.^(12)^

To ensure the model encompassed a variety of geometries, the training cases included a combination of 51 left‐ and 54 right‐sided lesions; 40 upper, 22 mid, and 43 lower lobe cases; 17 anterior, 45 posterior, and 43 midline lesions; and 43 central and 62 peripheral lesions. Central and peripheral lesions were classified according to RTOG criteria, in which lesions within 2 cm of the proximal bronchial tree were considered central.^(17)^ The mean PTV was $38.05\pm 30.75\;{\text{cm}}^{3}$ (range: 3.93–202.47 cm^3^).

### B. Model training

**Figure 1:**
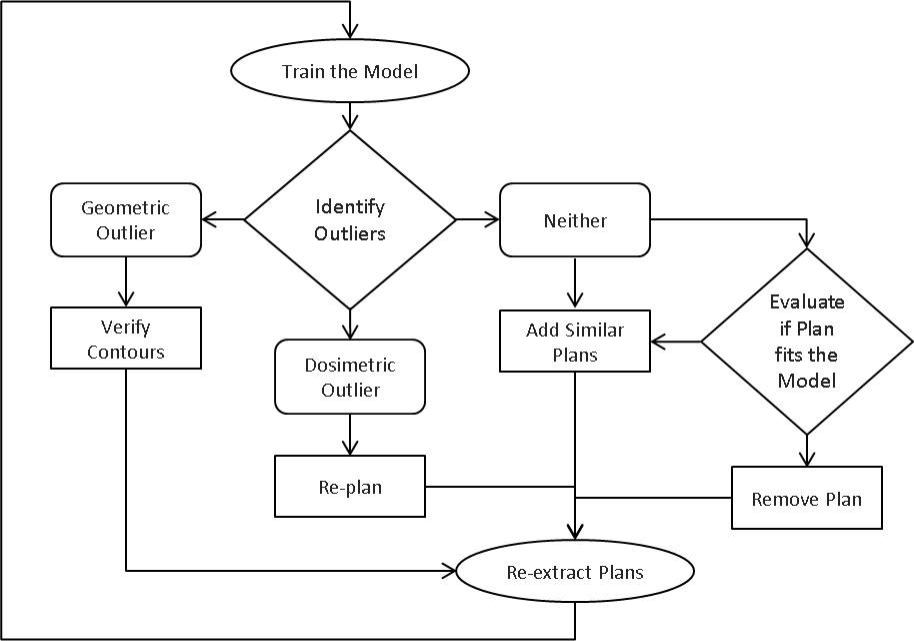
Workflow and decision tree of model training.

Training of the model consists of evaluating the model fit and pinpointing plans that are outliers in order to improve the model fit within the given training set. Outliers are individual training plans or structures that differ from the average of the training set. Model training is an iterative process, requiring several iterations to resolve outliers (Fig. 1). Through the combination of the statistical indices and regression analysis between the model generated DVH estimate and the plan dose for each training plan, plans or structures that are greater than two standard deviations from the average or considered to be influential data points in the model are identified as dosimetric or geometric outliers, respectively.^(13)^

A dosimetric outlier is a training plan with different dosimetric characteristics than what was estimated by the model for the same plan. For each training plan, the model generates an estimated DVH range. The midpoint within the estimated DVH range is compared to the DVH of the training plan and deviations are flagged as outliers. A common instance of a dosimetric outlier occurred when the model estimated OAR doses were superior to the original plan. In these instances the training plan was replanned to achieve better dose sparing. Other dosimetric outliers were found to be caused by suboptimal beam placements or atypical OAR tradeoffs in the training plan. These plans were replanned and the patient plan reextracted.

A geometric outlier is a structure with different geometric characteristics than the average structure in the KBM. A common instance of a geometric outlier in the model occurred when structures were found to be contoured incorrectly, deviating from the RTOG guidelines. This occurred for only a handful of the cases, where a slice was missing from the contour or the superior/inferior extent of the contour was too short. This was discovered by the difference in the shape of the OAR's DVH. Once the contour was fixed and verified by an attending physician, the data was reextracted with the new OAR contour.

If the plan was neither a geometric or dosimetric outlier, the plan was evaluated to determine its fit within the model. If the plan did not represent the typical geometry or case type of the model, such as a case with multiple or differentially dosed lesions, it was removed. However, if the plan was deemed to be a common clinical case, additional plans that were similar were added into the model to ensure that the scope of the model included all desired clinical cases.

After the addition or deletion of plans, all plan information was reextracted and used to rebuild and retrain the model. The OARs modeled in the KBM included the spinal cord, esophagus, heart, ribs, and normal lung (Total Lung–PTV). OARs such as the brachial plexus, stomach, and trachea that occurred less frequently clinically, were not modeled. In order to model an OAR, a minimum of 25 cases per OAR are needed.^(18)^

### C. Model validation

The model performance was validated on a validation plan set of 25 patient plans, independent from the training set. The patient geometries chosen in the validation plan set encompassed the range of geometries within the KBM. The validation plan set included a combination of 9 left‐sided lesions, 16 right‐sided lesions; 12 upper, 4 mid, and 9 lower lobe cases; 9 anterior and 16 posterior; and 10 central and 15 peripheral lesions. The mean PTV was $34.76\pm 30.10\;{\text{cm}}^{3}$ (range: 4.14–137.09 cm^3^). Each KBM was validated by applying it to the 25 validation plans and optimizing the plan with both IMRT and VMAT techniques. To avoid additional dosimetric variables during validation, the same beam arrangement initially designed by the dosimetrist in the clinical plan was used for the KBM validation. The optimization was run from start to finish with no planner intervention, including an intermediate dose calculation.^(19)^ All KBPs were renormalized so that the coverage to D95% of the PTV remained the same as the clinical plan.

**Figure 2:**
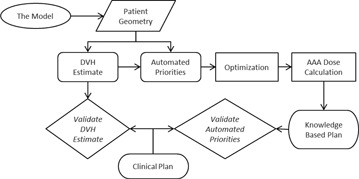
Workflow of KBM validation.

**Figure 3:**
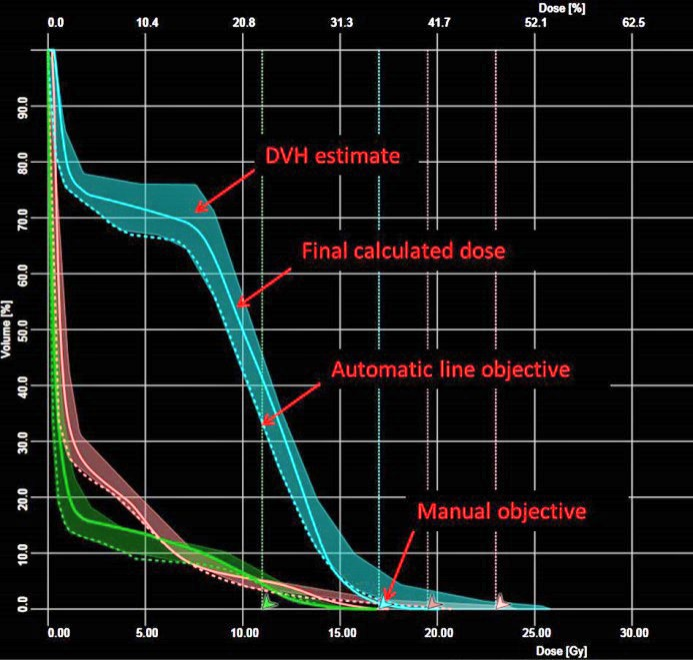
DVH of three oars: spinal cord (cyan), heart (orange), and esophagus,^(21)^ demonstrating the DVH estimate, final calculated dose after optimization, the automatic generated line objective, and a manual objective.

Model validation consists of two parts: validation of the DVH estimation and the automated priorities. Figure 2 outlines the workflow used to validate the KBM. The DVH estimate from the KBM is given as a range (Fig. 3). The DVH estimate is compared by visually inspecting that the shape is similar and the range encompasses the DVH of the clinical plan. Figure 3 shows an example of a DVH with the estimate and line objective overlaid on the clinical DVH. If any large discrepancies occur between the estimate and the clinical DVHs, the model is deemed to have had insufficient training.

The KBM uses the lower range of the DVH estimate as a line objective in the optimization, where the priority placed on the objective can be estimated by the KBM using the ratio between the weightings of the priorities of the PTV and the OARs. The automated priorities need to be validated to ensure that the weight relative to the PTV priority is sufficient so that OAR doses can be met without sacrificing PTV coverage. The automated priorities are validated by comparing the knowledge‐based plan (KBP) that was optimized with the automated priorities to the original plan and dose constraints. If the automated priority on the objective is sufficient, the KBP should achieve the line objective and meet OAR constraints. Manual objectives can also be included in the KBM to achieve OAR goals. In order to create a more flexible model that would be valid for other fractionation schemes, doses in the model were given in percentages of the prescription dose.

Dose points on the lower range of the DVH estimate were identified to evaluate the different models, for the modeled OARs: spinal cord, esophagus, and heart. The conformity index (CI) and gradient index (GI) were used to compare the plans. The CI is defined as the ratio between the volume of the prescription dose and the volume of the target, whereas the gradient index (GI) is defined as the ratio between the volume of 50% of the prescription dose and the volume of target. The paired Student's *t*‐test was used to compare the plans, where the threshold used for statistical significance was p<0.05.

### D. Tumor location

The geometrical distance between the target and OAR has been shown to correlate the amount of dose sparing achievable by an OAR.^(8)^ The location of lesions differs in lung treatments due to the multiple lobes and consequently affects the relative geometrical relationship between the PTV and the OARs. The ability of the KBM to accurately estimate the DVH can be altered if all clinically applicable geometries and locations are not included and trained in the model. Ideally, one KBM that encompasses all geometries would be more streamlined and easier to use in a clinical setting; however, submodels may better estimate DVHs for the OARs.

A total of five KBMs were created: a full KBM consisting of all lesion locations, central sub‐KBM (${\text{KBM}}_{C}$) consisting of centrally located lesions, peripheral sub‐KBM (${\text{KBM}}_{P}$) consisting of peripherally located lesions, anterior sub‐KBM (${\text{KBM}}_{\text{ANT}}$) consisting of anterior lesions, and posterior sub‐KBM (${\text{KBM}}_{\text{POST}}$) consisting of posterior lesions. The sub‐KBMs were compared to a full model (full KBM) to evaluate if it was necessary to create KBMs specific to the tumor location. To assess the differences between the sub‐KBMs, the KBMs were validated on three set of validation plans: the full validation set with 25 plans containing lesions from all locations, and two subvalidation sets separated based on lesion location.

**Figure 4:**
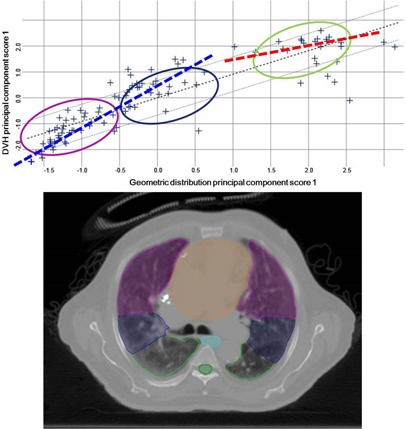
Spinal cord regression plot (top) showing three clusters corresponding to differing anterior to posterior locations in the lung (bottom). Magenta corresponds to anterior, blue corresponds to midline, and green corresponds to posterior. Dotted lines show expected correlation for the anterior and posterior sub‐KBMs.

The heart and esophagus structures are fairly midline relative to the lungs, whereas the spinal cord is located posteriorly. In a regression plot that correlates the geometric principal component score (PCS) to the DVH PCS, the spinal cord structure from all training plans exhibited three distinct clusters (Fig. 4). The three clusters correlate to different tumor locations: anterior (magenta), midline (navy), and posterior (green). By splitting the anterior and midline lesions from the posterior lesions, the expected correlation between the DVH PCS and the geometric distribution PCS for the two subgroups, shown in the dotted lines, changes drastically. Thus, two spinal cord sub‐KBMs were created to evaluate how the sub‐KBMs change the spinal cord DVH estimation. ${\text{KBM}}_{\text{ANT}}$ consists of anterior and midline lesions, which were included due to the relatively similar correlation between the PCSs, and ${\text{KBM}}_{\text{POST}}$ which consists of posterior lesions. The ${\text{KBM}}_{\text{ANT}}$ was trained with 71 cases and the ${\text{KBM}}_{\text{POST}}$ was trained with 34 cases. The full KBM, ${\text{KBM}}_{\text{ANT}}$, and ${\text{KBM}}_{\text{POST}}$ were validated with the full (25), anterior (9), and posterior (16) validation sets using an IMRT technique.

The model was split into central (${\text{KBM}}_{C}$) and peripheral (${\text{KBM}}_{P}$) models, where central and peripheral lesions were defined according to RTOG 0915 criteria. Lesions categorized as central are within or touching the proximal bronchial tree.^(17)^ The ${\text{KBM}}_{C}$ was trained with 43 cases, and the ${\text{KBM}}_{P}$ with 62 cases. The validation set was also split into central and peripheral sets, where the full KBM, ${\text{KBM}}_{C}$, and ${\text{KBM}}_{P}$ were validated on the full (25), central (10), and peripheral (15) validation sets using an IMRT technique.

### E. Dependency on beam arrangement

The RapidPlan software is an IMRT/VMAT optimizer that optimizes the modulation of the delivered radiation beam in order to deliver a desired dose distribution. The quantity and placement of the beams are defined by the planner prior to optimization. For a given patient geometry, the beam arrangement may vary from planner to planner, resulting in slightly different dose distributions. Beam angle optimizers have been used for IMRT treatment of non‐small cell lung cancer to find optimal beam arrangements to further decrease dose to OARs.^(20)^ The KBM takes into account the field geometry when estimating an OAR's DVH, by parsing areas within and outside the treatment field. Because beam placement can affect the dose distribution and suboptimal beam placements can make optimization more difficult, the full KBM was validated using two fixed‐beam IMRT templates in order to test the KBM's ability to estimate OAR DVHs and automated priorities. If a suboptimal beam arrangement occurs, where a beam traverses through an OAR, the automated priorities may not be sufficient to achieve an acceptable OAR dose.

Two fixed‐beam IMRT templates used to validate the KBM included a seven‐ and a nine‐field beam template. The seven‐field beam template arranged beams roughly 25°–30° apart. For a right‐sided tumor, the beam gantry angles were: 180°, 210°, 240°, 270°, 300°, 325°, 350°; and for a left‐sided tumor, the beam gantry angles were: 180°, 150°, 120°, 90°, 60°, 35°, 10°. The nine‐field beam template arranged beams approximately 20–25° apart. For a right‐sided tumor, the beam gantry angles were: 180°, 205°, 230°, 255°, 280°, 305°, 330°, 350°, 10°; and for a left‐sided tumor, the beam angles were: 180°, 155°, 130°, 105°, 80°, 55°, 30°, 10°, 350°. The nine‐field beam template covered a larger range compared to the seven‐field, and resulted in irradiating a larger portion of the heart and esophagus in a large majority of the validation cases.

Several studies have shown that the IMRT and VMAT techniques result in similar dose distribution. However for posterior lesions adjacent to the spinal cord, the majority of the cases at our institution are treated with posterior IMRT beams in order to obtain better conformity. VMAT delivery is limited by the machine limitation of the gantry unable to rotate past 180°. For posterior lesions, this limits the number of angles that a continuous arc can traverse. This can be bypassed if multiple posterior arcs are used; however, the VMAT delivery time increases as a result. Depending on the lesion location and difficulty of the case, VMAT can be used for posterior lesions. In order to support both IMRT and VMAT treatment techniques for posterior lesions in our model, additional VMAT posterior plans were added to the model to better train the model. The number of additional plans to add will vary from model to model, due to its dependence on the training set. The impact of any additional cases on the model can be gauged by assessing the regression plot and through revalidation of the new model.

## III. RESULTS

### A. DVH estimation and automatic priorities

During validation of the automatic priorities, the automatic priority on the line objective for several OARs was insufficient to achieve the dose constraint for the maximum point dose, defined as 0.035 cc. Several manual point objectives were added to the model in order to reduce the maximum point dose for OARs to within the tolerance dose. Table 2 shows the final model objectives used in the model, including whether the model‐generated DVH estimation automated priorities (KBMG) were used, or manual objective and priorities.

The 25 cases validated with the full KBM for both IMRT and VMAT techniques met all OAR constraints. Table 3 shows the IMRT and VMAT validation results of the full KBM and the lower DVH estimates. KBM lower estimates for the heart and spinal cord were less than the clinical plan, confirming that the DVH estimation of the KBM was successful. Compared to the clinical IMRT plans, the IMRT KBM resulted in lower doses to 1.2, 0.35, and 0.035 cc of the spinal cord by 0.5±0.9Gy (p=0.008), 0.8±1.2Gy (p=0.004), and 1.0±1.6Gy (p=0.006), respectively. The VMAT estimate for 0.035 cc of the esophagus was greater than the VMAT clinical plan and the IMRT estimate. The VMAT KBM resulted in higher doses to the maximum point dose of the esophagus by 1.1±2.2Gy, p=0.02. The CI between the clinical and KBP plans for both IMRT and VMAT were comparable. However, the GI increased in the IMRT KBPs compared to the clinical plans by 0.68±0.57, p=4.1E‐6, and increased in the VMAT KBPs by 0.20±0.34, p=0.009.

**Table 2:** Objectives and priorities used in the full model, including the objective type, the relative volume of the OAR in percent, the dose as a percent of the prescription dose, and the priority (unitless). The automatic objectives and priorities generated by the KBM are labeled as KBMG (knowledge‐based model‐generated), those with fixed numbers are manually added

*Structure*	*Objective type*	*Relative volume (%)*	*Dose (% of prescription)*	*Priority*
PTV	Upper	0	109%	100
	Lower	100	100%	100
	Lower	98	100.5%	100
Brachial Plexus	Upper	0	50%	80
Esophagus	Line	KBMG	KBMG	KBMG
	Upper	0	35%	60
Heart	Line	KBMG	KBMG	KBMG
	Upper	0	48%	80
Ribs	Line	KBMG	KBMG	30
	Upper	0	107%	0
Spinal Cord	Line	KBMG	KBMG	KBMG
	Upper	0	23%	75
Stomach	Upper	0	41%	80
Total Lung‐PTV	Upper	KBMG	20Gy	0
Trachea	Upper	0	71%	50

**Table 3:** Summary of OAR doses (Gy), CI, and GI from IMRT and VMAT validation, comparing the clinical plan and RapidPlan plan for 25 cases (mean±SD) (Gy) and DVH estimates from the full KBM

		*IMRT Validation*				*VMAT Validation*			
*Structure*	*Volume (cc)*	*Clinical (Gy)*	*IMRT Estimate (Gy)*	*IMRT KBP (Gy)*	*Difference KBP – Clinical (Gy)*	*Clinical (Gy)*	*VMAT Estimate (Gy)*	*VMAT KBP (Gy)*	*Difference KBP – Clinical (Gy)*
Esophagus	5	5.7±4.2	5.9±3.6	5.4±3.7	−0.3±1.3	5.9±3.7	5.9±3.7	6.0±3.6	0.2±1.7
	0.035	10.4±5.8	10.0±4.8	10.3±5.5	−0.0±1.6	10.1±5.1	12.0±6.0	11.0±5.0	1.1±2.2 ^a^
Heart	15	7.5±5.7	8.0±5.4	7.6±5.6	0.1±1.2	7.5±5.7	7.4±5.5	7.6±5.6	0.1±1.1
	0.035	13.2±9.9	10.5±7.6	13.3±9.9	0.1±1.6	12.5±9.3	12.4±9.0	12.6±9.3	0.1±0.8
Spinal Cord	1.2	7.6±2.9	7.3±2.8	7.1±2.5	−0.5±0.9 ^a^	7.2±3.1	7.0±2.8	7.1±2.8	−0.1±1.1
	0.35	8.6±3.4	8.2±3.3	7.8±2.9	−0.8±1.2 ^a^	7.9±3.4	7.8±3.3	7.8±3.0	−0.2±1.2
	0.035	9.4±4.0	8.8±3.5	8.4±3.3	−1.0±1.6 ^a^	8.7±3.9	8.7±3.5	8.4±3.3	−0.3±1.4
CI		1.02±0.05		1.00±0.03		0.99±0.03		1.01±0.03	
GI		4.8±0.8		5.46±0.78		4.52±0.85		4.77±0.99	

a Indicates a statistical difference (p<0.05) between the KBP and clinical plan.

### B. Tumor location

The final calculated doses for the esophagus, heart, and spinal cord from the five KBMs validated on full and subvalidation sets are shown in Table 4. When the model was divided according to central and peripheral locations and validated with the full validation set, compared to the full KBM, the ${\text{KBM}}_{P}$ resulted in lower doses to 0.035 and 5 cc of the esophagus by 0.4±0.8Gy (p=0.02) and 0.5±0.6Gy (p=0.003), respectively. When validated with the subset of peripheral tumors, the full KBM, ${\text{KBM}}_{C}$, and ${\text{KBM}}_{P}$ resulted in similar doses for all OARs. For the subset of central tumors, lower doses to 5 cc of the esophagus, by 0.5±0.6Gy (p=0.025), were observed in the ${\text{KBM}}_{P}$ compared to the full KBM

For the models divided according to anterior and posterior locations, ${\text{KBM}}_{\text{ANT}}$ resulted in lower doses to 0.035, 0.35, and 1.2 cc of the spinal cord by 0.2±0.4Gy for all dose points (p=0.02, 0.01, and 0.01, respectively), compared to the full KBM with the full validation set. Compared to the ${\text{KBM}}_{\text{POST}}$, the ${\text{KBM}}_{\text{ANT}}$ resulted in higher doses to 0.035 cc of the esophagus by 0.4±0.8Gy, p=0.02, as well as to 15 cc of the heart by 0.4±0.8Gy, p=0.03. Whereas compared to the ${\text{KBM}}_{\text{ANT}}$, the ${\text{KBM}}_{\text{POST}}$ resulted in higher doses to 0.035, 0.35, and 1.2 cc for the spinal cord by 0.3±0.6Gy (p=0.007), 0.4±0.6Gy (p=0.005), and 0.5±0.7Gy (p=0.003), respectively. When validated on the posterior validation subset, compared to the ${\text{KBM}}_{\text{ANT}}$, the ${\text{KBM}}_{\text{POST}}$ resulted in lower doses to 0.035 cc of the esophagus by 0.5±0.8Gy, p=0.01. In the anterior validation subset, the ${\text{KBM}}_{\text{ANT}}$ resulted in lower doses to 0.035, 0.35, and 1.2 cc of the spinal cord by 0.5±0.4Gy (p=0.01), 0.5±0.4Gy (p=0.009), and 0.5±0.5Gy (p=0.009), respectively, compared to the ${\text{KBM}}_{\text{POST}}$.

**Table 4:** Doses to esophagus, heart, and spinal cord from the full KBM and the four sub‐KBMs divided by tumor location validated on the full and partial validation sets

	*Validation Set*	*Esophagus*		*Heart*		*Spinal Cord*		
*Model*		*0.035 cc*	*5 cc*	*0.035 cc*	*15 cc*	*0.035 cc*	*0.35 cc*	*1.2 cc*
Full KBM	Full	10.3±5.5	5.4±3.7	13.3±9.9	7.6±5.6	8.4±3.3	7.8±2.9	7.1±2.5
${\text{KBM}}_{C}$	Full	10.2±5.5	5.4±3.8	13.0±9.6	7.4±5.4	8.4±3.3	7.8±2.9	7.1±2.6
${\text{KBM}}_{P}$	Full	10.0±5.3 ^a^	5.1±3.5 ^a^	13.1±9.6	7.3±5.6	8.3±3.4	7.7±3.0	6.9±2.6
${\text{KBM}}_{\text{ANT}}$	Full	10.6±5.8 ^b^	5.2±3.6	13.4±9.9	7.7±5.7 ^b^	8.2±3.3 ^a^, ^b^	7.6±2.9 ^a^, ^b^	6.8±2.6 ^a^, ^b^
${\text{KBM}}_{\text{POST}}$	Full	10.2±5.4 ^b^	5.3±3.8	13.0±9.6	7.3±5.4 ^b^	8.6±3.2 ^b^	8.0±2.9 ^b^	7.3±2.6 ^a^, ^b^
Full KBM	Central	14.0±5.0	8.0±2.5	17.8±9.8	9.5±5.8	10.4±3.0	9.5±2.6	8.6±2.3
${\text{KBM}}_{C}$	Central	13.9±5.3	8.1±2.8	17.3±8.9	9.2±5.6	10.4±3.1	9.6±2.8	8.7±2.5
${\text{KBM}}_{P}$	Central	13.6±4.8	7.4±2.6 ^a^	17.1±8.3	8.9±5.3	10.4±3.1	9.4±2.6	8.4±2.4
Full KBM	Peripheral	7.9±14.0	3.7±3.4	9.8±9.3	6.1±5.1	7.1±2.8	6.6±2.5	6.0±2.2
${\text{KBM}}_{C}$	Peripheral	7.7±18.9	3.6±3.3	9.7±9.0	6.0±5.1	7.1±2.7	6.6±2.4	6.0±2.2
${\text{KBM}}_{P}$	Peripheral	7.6±4.1	3.5±3.1	10.0±9.5	6.1±5.3	7.0±2.8	6.5±2.6	5.9±2.3
Full KBM	Anterior	6.1±2.8	2.9±2.2	12.3±12.0	7.2±6.6	6.0±2.0	5.7±1.8	5.3±1.6
${\text{KBM}}_{\text{ANT}}$	Anterior	6.1±2.6	2.8±2.8	12.7±12.1	7.2±6.6	5.7±1.9 ^b^	5.5±1.7 ^a^, ^b^	5.1±1.5 ^a^, ^b^
${\text{KBM}}_{\text{POST}}$	Anterior	5.9±2.4	2.5±1.9	11.4±11.1	6.5±6.0	6.2±2.0 ^a^, ^b^	6.0±1.9 ^a^, ^b^	5.6±1.8 ^a^, ^b^
Full KBM	Posterior	12.8±5.3	6.8±3.7	13.8±9.1	7.8±9.1	9.8±3.1	8.9±2.8	8.0±2.5
${\text{KBM}}_{\text{ANT}}$	Posterior	13.2±5.6 ^b^	6.6±3.5	13.9±13.8	8.0±5.4	9.6±3.1	8.7±2.7	7.8±2.5
${\text{KBM}}_{\text{POST}}$	Posterior	12.6±5.1 ^b^	6.9±3.7	13.8±9.1	7.7±5.2	9.9±3.0	9.1±2.7	8.2±2.5

a Indicates a statistically significant difference between the studied model and the full KBM model.

b Indicates a statistically significant difference between the studied model and the complementary model.

### C. Beam arrangement

During the initial phases of DVH estimation validation, the DVH estimates for the spinal cord and esophagus OARs for the VMAT technique for posterior lesions were found to be insufficient, resulting in estimates that were higher than the clinical plan. Upon conclusion of optimization and dose calculation, the spinal cord and esophagus structures in the KBM plan did not meet OAR constraints. However, plans validated using the IMRT technique had good estimates and were able to achieve OAR doses. The discrepancy in plan quality between the treatment techniques (IMRT vs. VMAT) was thought to be due to the differences in beam geometry, particularly for posterior lesions. After an additional five posterior VMAT lesions were added, for a total of 34 posterior cases in the full KBM, the DVH estimates were lowered to values within the clinical plan (Table 3).

For plans created with seven‐ and nine‐field templates, all OARs met the planning criteria. The nine‐field beam arrangement resulted in lower spinal cord doses, 6.7±2.9Gy vs. 7.3±3.0Gy (p=0.05), compared to the seven‐field arrangement. However, compared to the clinical plan, the nine‐field beam arrangement resulted in higher doses to 0.035 cc (14.5±10.1Gy vs. 13.3±9.8Gy, p=0.01) and 15 cc (8.5±6.5Gy vs. 7.6±5.6Gy, p=0.04) of the heart. The CI was comparable between the two template beam arrangements, however, the GI increased in the nine‐field plans from 5.9 to 6.1 compared to the seven‐field plans, p=0.0001, as well as compared to the clinical plans’ GI of 5.9, p=0.013.

## IV. DISCUSSION

In this study, a lung SBRT KBM was created using a commercial DVH algorithm and validated for use on a variety of lesion locations, planning technique, and beam arrangements. Previous studies of KBMs on prostate, head and neck, and hepatocellular cancers have shown good results compared to clinically treated plans.^9^, ^10^, ^11^, ^12^ Less variability in beam arrangements and planning techniques are often seen in head and neck and prostate treatments, either using a seven‐ or nine‐field static IMRT evenly spread out over 360° or a full arc VMAT technique. A variety of planning techniques are often used in lung SBRT treatments, and thus the model created in this study was evaluated to verify the robustness of the KBM, as well as for the clinical needs at our institution.

Often in SBRT, in addition to dosimetric constraints on volumetric percentages of the OARs, constraints are warranted for the maximum point dose of an OAR. During validation of the KBM, multiple KBPs did not meet the maximum point dose for multiple OARs. This was resolved by placing a manual objective and priority below the maximum allowable OAR dose. The KBM was revalidated to ensure the manual priority placed on the objective was adequate for differing patient geometries. For cases where the PTV abuts the OAR, the OAR maximum dose could not be met running the model independently. In order to achieve the OAR dose, several rounds of optimization with planner intervention were necessary. As a result, PTV coverage is often sacrificed in order to meet the OAR constraint. Although the model was trained with cases where the PTV abuts the OAR, this may be a limitation of the KBM algorithm. Furthermore, because the model was created based on the contouring criteria at our institution, where the PTV was manually modified to avoid overlap with the OARs, the principal component employed in this KBM is unable to model situations in which the OARs overlap with PTVs. This may limit the types of cases that the KBM can be applied to and may not be applicable to institutions that do not use the same contouring criteria. For complex cases, the benefit in efficiency of the KBP may be limited. Future studies include training the KBM with more difficult and complex cases to test if the model can learn to produce doses similar to those down with manual planning.

The full KBM was validated using both IMRT and VMAT techniques and resulted in fairly equivalent OAR doses with clinical IMRT and VMAT plans. Although more studies need to be performed, preliminary results of the validation plans show that the KBM can quickly achieve a plan that is near equivalent in regard to OAR dose to the clinical plan with only one optimization. In the case of the KBM validated on IMRT, the KBPs resulted in better OAR dose for the spinal cord compared to the clinical plan. However, the VMAT KBP resulted in higher doses to the esophagus. This may be due to the difference in beam geometry between IMRT and VMAT, where the gantry rotation is limited by machine. In the model training, more posterior VMAT plans were added to the model to compensate for the higher spinal cord dose, which may have resulted in a tradeoff between the spinal cord and esophagus doses in the model impacting the other lesion locations. It should be noted that, even though the OAR doses met the criteria, the dose distribution should be visually inspected to ensure dose conformity. The CIs for all plans were all very near an ideal CI of 1, however larger deviations occurred amongst the GIs. Both VMAT and IMRT KBPs resulted in larger GIs than the clinical plans. This may have been a result of the KBM optimizing the OAR doses too aggressively, resulting in a slightly skewed planar dose distributions, as well as the clinical plan being optimized using multiple iterations in conjunction with different planning techniques, such as optimization structures, to achieve a smaller gradient index.

When independent anterior and posterior KBMs were created, the ${\text{KBM}}_{\text{ANT}}$ achieved better doses to the spinal cord compared to the full KBM, whereas the ${\text{KBM}}_{\text{POST}}$ achieved better doses to the esophagus and heart compared to the ${\text{KBM}}_{\text{ANT}}$. Similarly for the central and peripheral models, the ${\text{KBM}}_{P}$ achieved better results for the esophagus than the ${\text{KBM}}_{C}$. Originally it was thought that a posterior model would result in better spinal cord doses, and the central model would achieve better esophagus and heart doses due to the proximity of the PTV location to the OARs. Previous studies comparing three head and neck models show that the resulting OAR doses depend heavily on the type of cases that the model was trained with.^(14)^ In our study, the sub‐KBM where the distance between the OAR and PTV was smaller did not achieve lower doses to the OAR. The geometric PCAs for those plans may have been in the outer range of the model, and extrapolated for geometries where the distance between the OAR and PTV was large. In this study, the plans in the submodels were not replanned. This may have resulted in greater differences between the sub‐KBMs; however, replanning all the training cases would have changed the training set drastically. Overall, the results of validation showed similar tradeoffs in OAR doses between the ${\text{KBM}}_{\text{ANT}}$ and ${\text{KBM}}_{\text{POST}}$ models and the ${\text{KBM}}_{C}$ and ${\text{KBM}}_{P}$ models. In the full KBM, all OARs benefit in the combined model. Differences between the full KBM and the sub‐KBMs were minimal, less than 1 Gy, which may be clinically insignificant.

When using beam templates, KBPs met all OAR constraints, although slight tradeoffs between the spinal cord and heart were observed. This may be due to the beam arrangements in the clinical plans which were purposely arranged so that beams did not enter through the cord or the heart. The optimizer and automated priorities were able to achieve OAR doses; however, with suboptimal beam placement, an increase in dose to OARs may be expected. Furthermore, the use of beam templates is common practice in many clinical settings. The combination of KBM and beam templates may allow for increased efficiency and throughput of plans.

## V. CONCLUSIONS

RapidPlan KBMs can be used to generate lung SBRT plans that are comparable to clinical plans. With sufficient model training and variability of patient geometry in the model, a KBM can be created to encompass both IMRT and VMAT techniques, differing tumor locations, and template beam arrangements.

## ACKNOWLEDGMENTS

The authors would like to thank Helen Phillips and Esa Kuusela for their technical assistance and support in creating the lung SBRT model, and Siming Lu for his help with plan validation.

## COPYRIGHT

This work is licensed under a Creative Commons Attribution 3.0 Unported License.
